# Supplementary material for: Newborn body composition after maternal bariatric surgery
Source: PLoS One. 2020 May 13;15(5):e0231579. doi: 10.1371/journal.pone.0231579 (PMC7219732; doi:10.1371/journal.pone.0231579)
Supplement: Attachment — Submitted filename: PLOS_one answer to reviewers_final.docx [file pone.0231579.s001.docx]

**Editor Comments**

General:
- I understand that the data cannot be made publicly available. However, at least the analysis code could be put online, e.g. at [www.osf.io](http://www.osf.io/), with the exact site being mentioned in the manuscript.

First of all: Thank you for a detailed review of our manuscript that certainly improved the quality of our work.

We are, unfortunately not, familiar with the website mentioned. The analysis will be available by contacting the corresponding author. Please, refer to comment under acknowledgement.

- Please provide a STROBE statement and adjust the manuscript accordingly where necessary.
Strobe statement is added, and the checklist is attached (Appendix 1).

Methods:
- Were there any exclusion criteria for the RYGB group regarding e.g. maximum time between RYGB and birth or intermittent pregnancies?

No exclusions, all pregnancies were included after informed oral and written consent was retrieved.

- Was it checked whether any of the reference mothers had had RYGB previously?

Yes, there was no woman in the reference material that had undergone bariatric surgery of any kind, it has been added to the methods section.

- How were SGA and LGA calculated?

Sorry for not making this clear. We used Marsal’s reference, which is the reference used by Danish neonatologists (and used in most Nordic countries). This is more clearly stated in the manuscript now, please see line 118-119.

- l.140-152: This approach is quite unusual. Why did the authors not simply calculate regression models on all mothers and report the regression coefficient for RYGB as a binary variable?
We chose this approach since it was the only way we could estimate the effect of weight loss induced by surgery and the effect of bariatric surgery on foetal growth during pregnancy.

A linear regression, with RYGB as a binary variable, has been calculated and added as Appendix 2 to this review and is of course also informative.

We, however, believe that the analyses we made are better to illustrate the enormous effect bariatric surgery has on fetal growth since it is this and not the weight loss that induce the primary changes. We find it interesting to see that the women that had undergone bariatric surgery has the same mean gestational weight gain and there is also no difference I placental weight. Despite this, their offspring is significantly smaller.

Results:
- Figure 1 should be described in more detail in the main text. Did the RYGB mothers who were excluded differ from those included in certain characteristics?

We did not collect data on the women who gave birth at another hospital (n=13). There was no difference in baseline maternal characteristic in the women that gave birth premature or the got admitted to NICU (n=7). This also added to the text.

- Figure legends should be presented below the figures, not in the main text.

It has been changed.

- Table 1: Suggest to provide all p-values, also the NS ones. The authors should report n(%) for all categorical variables. The chi-square test is not appropriate for maternal smoking, SGA/AGA/LGA and birth weight due to too low expected cell numbers.

All p-values are now added as well as n(%) for all categorical values. The Chi-square test for maternal smoking, SGA/AGA/LGA and birthweight has been removed.

- Table 2: Please provide 95% confidence intervals and state more clearly how the p-values were calculated.

The 95 CI% and SD are added. We have tried to clarify how the p-values were calculated.

Discussion:
- l.183/184: The preterm rate in the control children seemed unusually low. What could be a potential explanation for this low rate?

We cannot explain the low rate of prematurity in the control children and we were also surprised by this finding.

- l.265-268: Could the exclusion of the RYGB preterm children have biased the main findings? It may be worth to include them in a sensitivity analysis which could also be adjusted for preterm birth.

We did not dexa-scan the premature born children. The incidence of growth restriction is significantly higher in the premature born babies and our aim was to examine how term newborn body composition was affected by bariatric surgery. Therefore, women with pre-eclampsia were also excluded.

- Conclusion (and title): Wording implying causation should be avoided in cross-sectional studies.

Sorry, the title has been changed and the wording in the conclusion has been changed.

**Reviewer #1**:
Thank you for the opportunity to review this manuscript. This is a Danish study on offspring body composition after bariatric surgery compared with control births. In general, this is a very interesting and well conducted study of importance for the research field.
General comments

Thank you for reviewing our article, which improved our work.
1. How was the model for expected values of birth weight developed? Does it use the Marsal curves as reference? Is it appropriate to use gestational weight gain in the model given that this could be a mediator between RYBG and the outcome under study? Could the model be overfitted?

We calculated expected values using our reference material. We used linear regression. For each woman, expected values for birth weight and body composition compartments were calculated based on the pre-surgery and pre-pregnancy BMI. The expected value was compared to the actual measured value. We used the actual measured gestational weight gain (GWG).

As an example, we show you the regression used to calculate birth weight (this is also presented in the statistics section):

Expected birthweight = (- 3086.1)+ (pre-preganancy BMI or pre-surgery BMI*13.8)+(GWG*22.9)+(days of gestation at birth*22.3)+(if primiparity -192)+(if female sex -63.8).

It is important to notice that there was no difference in mean GWG comparing the RYGB women with the reference material and we do not believe this mediate the change in birth weight and newborn body composition since the RYGB women delivered smaller babies despite an appropriate mean GWG. One should also notice that there is no difference in mean placental weight.

We have tried to clarify our method under the statistics section.

2. The study included both obese and normal weight comparators. Is there a risk that the comparison would not be optimal? Would a matched analysis be superior where women of the same BMI would be compared?

We considered a matched analysis when we started the work. We believe the method used is appropriate, using linear regression we calculate an expected outcome in the reference material based on BMI, GWG, gestational age at birth, parity and newborn sex. The problems with a matched analysis would be to obtain enough controls with high pre-pregnancy BMI, few women have pre-pregnancy BMI of 44, uncomplicated pregnancy and uneventful delivery. The mean pre-surgery BMI was 44.0.

3. How was the attribution of the average reduction in new-born free fat mass calculated?

We have tried to explain this in the statistics section and clarify it in the results section. Sorry for not doing it properly. The difference in the compartments between pre-surgery and pre-pregnacy BMI were compared to actual measured values. We concluded that the difference between the actual measured value and the estimated value based on the pre-pregnancy BMI was induced by the effect of RYGB during pregnancy. The babies are smaller and leaner than expected by the maternal pre-pregnancy BMI and GWG.

4. Almost half of the study participants were excluded and did not perform the DXA analysis. Did they differ in maternal and neonatal characteristics including birth weight for gestational age? Could this have influenced the findings?

Please see the answer to the editors question above. We did not obtain neonatal characteristics on excluded infants. We would guess that a proportion of babies delivered premature have lower birth weight SDS. Including them, might have done the differences more pronounced.

5. Were there any missing data for the variables included in the study? If yes, how was this handled in the analysis?

We only included women for whom the offspring had a successful dexa-scan. The data are therefore complete.

Specific comments
1. Please include proportions (%) in Table 1 for size for gestational age and birthweight.

Proportions is now included.

2. In Table 1, was it weight loss from surgery to conception or birth? Please include this for the variable.

It was until conception, and this is now included.

3. In Table 2, include as a footnote how the expected measures were obtained.

The footnote now include how expected values were obtained.

4. Please exclude paired t-test from the title of Table 2.

Paired t-test is removed from title and added to footnote.

5. Could not early pregnancy weight be retrieved from the antenatal medical record instead of having it self-reported?

In Denmark women do not visit their midwife/doctor until gestational week 8-10, therefore we have self-reported weight. This pre-pregnancy weight is also self-reported in the reference material.

6. The discussion on the biological reason for the results could be further developed. What metabolic, nutritional and hormonal differences could induce such an effect?
Based on current literature I am not sure we can further develop this, but if there are any reference we have not included that are relevant, we would like to do so.

**Reviewer #2:**

In this manuscript, Malchau Carlsen et al. compared newborn's body composition measured by DXA within 48 h of birth in neonates born after maternal bariatric surgery (RYGB) (N=25) with measures obtained from a reference sample of newborns from obese and normal-weight mothers (similar pre-pregnancy BMI). They found that neonates born after maternal RYGB had a lower birthweight, a lower fat-free mass, lower fat mass, lower fat percentage, and a lower part of the fat distributed abdominally than neonates from the reference sample.
This study reports novel data, and answers a clinically relevant question, that has not been assessed previously in litterature. Whereas the sample size is rather small (N=25) for the post- bariatric surgery sample, the study uses standard measurements of newborn's body composition.

Thank you for considerate input, which have improved our paper.

Major:
1. The selection of the "reference material" is not clearly explained. Some details are not described in the method and are important for comprehension and interpretation of the data: How the mothers in reference sample were recruited and how mothers included in this specific analysis were selected ? Were the procedures exactly the same in the two studies? Was the reference cohort matched for any characteristics of the RYGB cohort?
The reference material was recruited in the same manner as the RYGB women. The reference cohort was not matched in any way. The reference material was recruited before the RYGB cohort. For further detail please read reference 13 and 15.

2. I suggest that the statistical analysis should be reviewed by a statistician, as I am not in measure to assess the quality of the analysis proposed. The interpretation of the results of the expected body fat mass based on previously described associations as a % contribution from each variable needs to be commented by a statistician as it may not be appropriate to interpret directly as the contribution of a variable to the outcome. As this is an important message from the manuscript, interpretation of these models should be done with caution.
We have consulted the statistician at our hospital, but any external review would also be welcome. Please see answer to reviewer 1 first comment.

3. The authors should explain the sample size determination for both groups.

We wanted to describe differences in birth weight and body composition after bariatric surgery, and there is no previous data on this, therefore sample size calculation was difficult.

Minor:
Line 153: RYGB instead of RYGBP
Thank you, it has been changed.

Line 199-200: these interpretation of data should be consider in the discussion rather than results section.

It has been deleted.

Line 220-223: a reference for glucose metabolism and hormonal alteration's impact on body fat mass would be appreciated

We have added two references.

**Appendix 1**

STROBE Statement—Checklist of items that should be included in reports of ***cohort studies***

|  | Item No | Recommendation |
| --- | --- | --- |
| **Title and abstract** | 1 | (*a*) Indicate the study’s design with a commonly used term in the title or the abstract |
|  |  | (*b*) Provide in the abstract an informative and balanced summary of what was done and what was found |
| Introduction | | |
| Background/rationale | 2 | Explain the scientific background and rationale for the investigation being reported |
| Objectives | 3 | State specific objectives, including any prespecified hypotheses |
| Methods | | |
| Study design | 4 | Present key elements of study design early in the paper |
| Setting | 5 | Describe the setting, locations, and relevant dates, including periods of recruitment, exposure, follow-up, and data collection |
| Participants | 6 | (*a*) Give the eligibility criteria, and the sources and methods of selection of participants. Describe methods of follow-up |
|  |  | (*b*) For matched studies, give matching criteria and number of exposed and unexposed |
| Variables | 7 | Clearly define all outcomes, exposures, predictors, potential confounders, and effect modifiers. Give diagnostic criteria, if applicable |
| Data sources/ measurement | 8* | For each variable of interest, give sources of data and details of methods of assessment (measurement). Describe comparability of assessment methods if there is more than one group |
| Bias | 9 | Describe any efforts to address potential sources of bias |
| Study size | 10 | Explain how the study size was arrived at |
| Quantitative variables | 11 | Explain how quantitative variables were handled in the analyses. If applicable, describe which groupings were chosen and why |
| Statistical methods | 12 | (*a*) Describe all statistical methods, including those used to control for confounding |
|  |  | (*b*) Describe any methods used to examine subgroups and interactions |
|  |  | (*c*) Explain how missing data were addressed |
|  |  | (*d*) If applicable, explain how loss to follow-up was addressed |
|  |  | (*e*) Describe any sensitivity analyses |
| Results | | |
| Participants | 13* | (a) Report numbers of individuals at each stage of study—eg numbers potentially eligible, examined for eligibility, confirmed eligible, included in the study, completing follow-up, and analysed |
|  |  | (b) Give reasons for non-participation at each stage |
|  |  | (c) Consider use of a flow diagram |
| Descriptive data | 14* | (a) Give characteristics of study participants (eg demographic, clinical, social) and information on exposures and potential confounders |
|  |  | (b) Indicate number of participants with missing data for each variable of interest |
|  |  | (c) Summarise follow-up time (eg, average and total amount) |
| Outcome data | 15* | Report numbers of outcome events or summary measures over time |
| Main results | 16 | (*a*) Give unadjusted estimates and, if applicable, confounder-adjusted estimates and their precision (eg, 95% confidence interval). Make clear which confounders were adjusted for and why they were included |
|  |  | (*b*) Report category boundaries when continuous variables were categorized |
|  |  | (*c*) If relevant, consider translating estimates of relative risk into absolute risk for a meaningful time period |
| Other analyses | 17 | Report other analyses done—eg analyses of subgroups and interactions, and sensitivity analyses |
| Discussion | | |
| Key results | 18 | Summarise key results with reference to study objectives |
| Limitations | 19 | Discuss limitations of the study, taking into account sources of potential bias or imprecision. Discuss both direction and magnitude of any potential bias |
| Interpretation | 20 | Give a cautious overall interpretation of results considering objectives, limitations, multiplicity of analyses, results from similar studies, and other relevant evidence |
| Generalisability | 21 | Discuss the generalisability (external validity) of the study results |
| Other information | | |
| Funding | 22 | Give the source of funding and the role of the funders for the present study and, if applicable, for the original study on which the present article is based |

*Give information separately for exposed and unexposed groups.

**Note:** An Explanation and Elaboration article discusses each checklist item and gives methodological background and published examples of transparent reporting. The STROBE checklist is best used in conjunction with this article (freely available on the Web sites of PLoS Medicine at http://www.plosmedicine.org/, Annals of Internal Medicine at http://www.annals.org/, and Epidemiology at http://www.epidem.com/). Information on the STROBE Initiative is available at http://www.strobe-statement.org.

**Appendix 2**

**Dependent variable: Offspring birth weight (g)**

| **Coefficients^a^** | | | | | | |
| --- | --- | --- | --- | --- | --- | --- |
| Model | | Unstandardized Coefficients | | Standardized Coefficients | t | Sig. |
|  |  | B | Std. Error | Beta |  |  |
| 1 | (Constant) | -2961,551 | 868,989 |  | -3,408 | ,001 |
|  | RYGBPy_n | -297,680 | 97,409 | -,151 | -3,056 | ,002 |
|  | Gestational age (days) | 21,532 | 2,892 | ,373 | 7,445 | ,000 |
|  | Pre-pregnancy BMI | 14,204 | 4,402 | ,169 | 3,227 | ,001 |
|  | GWG | 23,275 | 4,336 | ,282 | 5,368 | ,000 |
|  | Offspring sex | -81,525 | 51,163 | -,078 | -1,593 | ,112 |
|  | Maternal age | -3,665 | 5,840 | -,032 | -,627 | ,531 |
|  | Primiparity y_n | -216,089 | 55,439 | -,203 | -3,898 | ,000 |
|  | Smoking y_n | 112,861 | 85,543 | ,066 | 1,319 | ,188 |
|  | | | | | | |

**Dependent variable: Offspring fat-free mass (g)**

| **Coefficients^a^** | | | | | | |
| --- | --- | --- | --- | --- | --- | --- |
| Model | | Unstandardized Coefficients | | Standardized Coefficients | t | Sig. |
|  |  | B | Std. Error | Beta |  |  |
| 1 | (Constant) | -1335,424 | 654,876 |  | -2,039 | ,042 |
|  | RYGBPy_n | -253,381 | 73,408 | -,168 | -3,452 | ,001 |
|  | Gestational age (days) | 16,998 | 2,180 | ,386 | 7,799 | ,000 |
|  | Pre-pregnancy BMI | 1,625 | 3,317 | ,025 | ,490 | ,625 |
|  | GWG | 13,121 | 3,267 | ,208 | 4,016 | ,000 |
|  | Offspring sex | -177,831 | 38,557 | -,223 | -4,612 | ,000 |
|  | Maternal age | -1,818 | 4,401 | -,021 | -,413 | ,680 |
|  | Primiparity y_n | -180,040 | 41,779 | -,222 | -4,309 | ,000 |
|  | Smoking y_n | 71,871 | 64,466 | ,055 | 1,115 | ,266 |
|  | | | | | | |

**Dependent variable: Offspring body fat (%)**

| **Coefficients** | | | | | | |
| --- | --- | --- | --- | --- | --- | --- |
| Model | | Unstandardized Coefficients | | Standardized Coefficients | t | Sig. |
|  |  | B | Std. Error | Beta |  |  |
| 1 | (Constant) | -22,933 | 7,293 |  | -3,145 | ,002 |
|  | RYGBPy_n | -2,500 | ,818 | -,154 | -3,058 | ,002 |
|  | Gestational age (days) | ,078 | ,024 | ,164 | 3,211 | ,001 |
|  | Pre-pregnancy BMI | ,195 | ,037 | ,282 | 5,273 | ,000 |
|  | GWG | ,199 | ,036 | ,293 | 5,471 | ,000 |
|  | Offspring sex | 2,071 | ,429 | ,241 | 4,823 | ,000 |
|  | Maternal age | -,033 | ,049 | -,035 | -,681 | ,496 |
|  | Primiparity y_n | -,090 | ,465 | -,010 | -,193 | ,847 |
|  | Smoking y_n | ,745 | ,718 | ,053 | 1,037 | ,300 |
