# Supplementary material for: Newborn body composition after maternal bariatric surgery
Source: PLoS One. 2020 May 13;15(5):e0231579. doi: 10.1371/journal.pone.0231579 (PMC7219732; doi:10.1371/journal.pone.0231579)
Supplement: Attachment — Submitted filename: Answer to second reviewRYGB_final.docx [file pone.0231579.s002.docx]

The authors did well to revise their manuscript in response to most of my questions. However, a few issues remain:

- l.197-205: These results should be described in much more detail. In particular, the birthweight findings should be mentioned, and the calculations of how much impact the surgery vs. weight loss had should be explained more clearly. Besides, I do not understand the authors' conclusion that 2% of the reduction in fat free mass could be attributed to weight loss, because the estimated difference based on pre-surgery compared to pre-pregnancy BMI was 5 g lower and not higher.

Thank you for your very useful comments, we are sorry we haven’t made this clear during the first review. We have tried to make it clearer in the text and hope we now have improved the description.

We looked at the difference in our estimates. Pre-surgery BMI estimates for mean fat-free mass was 3345 g, pre-pregnancy BMI estimates for fat-free mass 3340 g, the difference is hence 5 g, the effect of weight loss before pregnancy. The difference between pre-surgery fat-free mass and actual measured fat-free mass (3345 g - 3062 g) is 283 g. Therefore, weight loss only accounts for a small reduction in fat-free 5 g/283 g = 2%. The effect of bariatric surgery during pregnancy (276g/283g) induced the greatest change.

- l.209: Also the third column shows 95% CIs.

That is correct.

- I am still in doubt the analyses presented in table 2 are sound. In particular, I am wondering whether the authors took the expected values as fixed, which would mean that the 95% CIs for the differences between expected and measured values did not take the uncertainty in the estimation of the expected values into account. The authors should clarify this and/or revise their analyses accordingly.

Good point. We did not use the values as fixed, so the uncertainty is included in the 95%CI, which also are rather wide.

- The results from Appendix 2 should be presented in suitable fashion, i.e. as scientific tables with explanations, and the results should also be mentioned in the main text and set into context to the results from Table 2.

The results from appendix 2 are incorporated in our text now, and presented in a new table 2. The old table 2 is renamed table 3. The findings in table 2 are in line with table 3 (difference between pre-pregnancy BMI values and actual measured values).

- The authors should provide a statement in the main text why the analysis code cannot be made publicly available.
A short statement has been added. Please see line 320.
